# The impact of aging on acute coronary syndromes: an EHR-based analysis

**DOI:** 10.1007/s12471-025-02003-9

**Published:** 2025-12-01

**Authors:** Quinten P. Hoogervorst, Charlotte E. P. Siegers, Jan van Ramshorst, Maurits T. Dirksen, Ton A. C. M. Heestermans, Olivier Drexhage, Victor A. W. M. Umans

**Affiliations:** Department of Cardiology, Noordwest Ziekenhuisgroep, Alkmaar, The Netherlands

**Keywords:** Octogenarians, Nonagenarians, ACS, PCI

## Abstract

**Introduction:**

The number of octo and nonagenarians presenting with acute coronary syndrome (ACS) is rising and underreported. Therefore, this study aims to clarify patient characteristics and compare outcomes of an initial invasive strategy versus optimal medical treatment.

**Methods:**

All consecutive ACS patients from 2020 until 2023 were admitted, and with EHR data extracted. Multi-variation analyses were carried out in three age groups: 80–84, 85–89 and 90+.

**Results:**

A total of 1,036 consecutive patients over 80 years old were analyzed. A predominance of women, lower rates of angiography/PCI and hypercholesterolemia were observed in the nonagenarians. CABG was only performed in the 80–84 yrs group. No differences in the complication rates (type 3a bleeding, CVA, or secondary ICU admission) between the invasive and OMT group at any age. At 3 months, a trend towards a better outcome in all-cause mortality was seen in the invasive group in the age groups: 80–84: HR 0.44 (0.19–1.04) (*p* = 0.06), 85–89: HR 0.46 (0.20–1.07) (*p* = 0.07) and significant better in 90+: HR 0.16 (0.03–0.85) (*p* = 0.03).

**Conclusion:**

In this consecutive cohort of 6,168 ACS patients, 1,036 (17%) were octo- and nonagenarians. Nonagenarians differ compared to 80+ and 85+ patients. At 30 days, mortality rates were 4% in the 80–84 group, 10% in the 85–89 group (*p* = < 0.001), and 15% in the nonagenarians (*p* < 0.001). This all-comer single-center study shows that appropriate selection may be feasible for an invasive strategy in ACS octo and nonagenarians in terms of safety and outcome.

## What’s new?

- In ACS all-comers, 17% are octo and nonagenarian patients.
- At 30 days, mortality rate differs within the 2 octogenarian groups: 4% in the 80–84 group and 10% in the 85–89 group and rising to 15% in the nonagenarians.
- This single center study shows that physicians are able to appropriately select ACS octo and nonagenarians for an invasive strategy.
- An invasive strategy in elderly is safe and has a favorable outcome, mostly seen in the NSTEMI.

## Introduction

With increasing life expectancy in the Western world, the number of octogenarians and nonagenarians is rising, leading to a larger group of elderly patients with acute coronary syndrome (ACS) in these age groups [1]. ACS is common in the elderly and has a major impact on their morbidity, mortality, and quality of life [2]. Treating ACS in octogenarians and nonagenarians is challenging due to multimorbidity, frailty, atypical presentation, and lack of evidence-based treatment options [2].

Current ESC ACS guidelines (2023) recommend a holistic strategy for the “very elderly NSTEMI and UAP patient” due to lack of robust evidence. For STEMI patients, the guidelines recommend primary PCI if possible. Octogenarians and nonagenarians are underrepresented in clinical studies; only a few small studies investigated mortality outcomes of invasive versus conservative therapy [3].

Aging is an important risk factor and determinant of outcome, although randomized trials in octogenarians and invasive strategies are only recent and scarce. Three trials have addressed such outcomes: the Italian Elderly ACS included 313 patients over 75 years [4], the coMOrbilidades en el Síndrome Coronario Agudo (MOSCA) trial included 106 patients over 70 years [5] and the After-Eighty study [6] included 457 octogenarians. Of these, the latter showed a superior outcome in the invasive treated patients. However, efficacy decreased with increasing age and was not significant in patients over 90 years. Specific to NSTEMI patients the recent Senior Rita study of Kunadian et al. (2024) found no significant difference in cardiovascular death between invasive vs conservative treatment [7].

Finally, the observational study of Gimbel et al. found a beneficial effect of PCI and CABG in ACS patients above 75 of age compared to the pharmacologically treated group [8].

Mortality rates are higher in the octogenarian and nonagenarian population [1, 9, 10] and outcomes of treatment options are not unequivocal. Therefore, this study compares real-world ACS outcomes between an initial invasive treatment and optimal medical treatment (OMT) strategy in consecutive patients over 80 years in whom therapy selection was based on frailty by clinical judgement.

## Methods

### Data extraction

During the period 2020–2023, all consecutive patients experiencing ACS who were admitted to the Northwest hospital group (NWZ) were included in this analysis. Approval was given by the local ethics commission. Data was collected from the EPD using extraction scripts to gain physician-entered clinical data on the 80+ group with DBC diagnosis STEMI, NSTEMI, and unstable angina pectoris.

STEMI patients were on presentation eligible for primary PCI conform the guideline, which does not discriminate by age. However, patients who had cognitive impairment, nursing home residents, had a known severely reduced LV function, or renal insufficiency were first clinically screened at arrival. In NSTEMI patients, we additionally incorporated eGFR, Hb, and CRP laboratory results in the work-up to intervention. A geriatric assessment was not performed routinely.

Data on medical (cardiac) history and risk factors were already recorded in specific EPD sections in most patients. Patients with missing data in the cardiac medical history, risk factors, or a missing BRP-mortality check were excluded.

The primary outcome was all-cause mortality at 90 days.

A PCI is defined as either a successful or unsuccessful PCI performed during admission. A CABG is defined as a CABG procedure conducted within 30 days following admission, without a preceding PCI.

BARC type 3a bleeding was determined by a hemoglobin decrease of ≥ 1.86 mmol/L [9].

In-hospital mortality was checked by the registration in the EHR. Mortality after discharge was controlled by BRP check.

### Statistical analysis

SPSS statistics 28 (SPSS Inc., Chicago, IL, USA) was used for statistical analyses. Continuous variables are presented as mean and standard deviation (SD). Normal distribution is tested by the Shapiro-Wilk test of normality. When not normally distributed, the Mann-Whitney test for two groups and the independent-samples Kruskal-Wallis test for 3 groups were applied. Chi-square tests were used for dichotomic outcomes. Multivariate analyses of 90-day mortality were conducted by logistic regression. Cox-regression analyses were used for survival analyses and included all patients whom had all variables known: sex, history of MI, history of PCI, history of CABG, smoking, hypertension, hypercholesterolemia, DM, type of ACS, and those with available Hb lowest levels, eGFR highest result during admission, CRP levels highest result during admission and troponin levels highest result during admission.

## Results

**Fig. 1:**
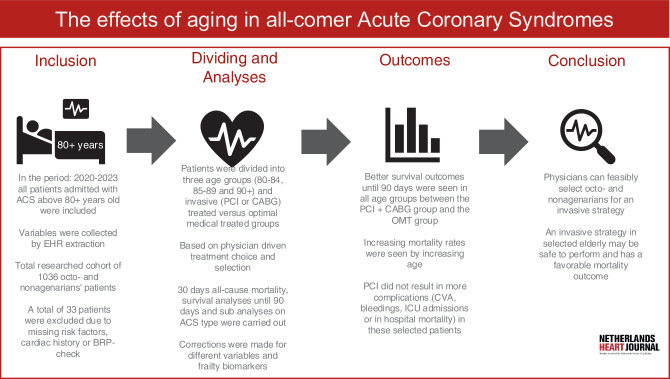
Infographic The effects of aging in all-comer Acute Coronary Syndromes

In 4 years 6168 all-comers consecutive ACS patients were admitted. Of these, 1069 (17%) were octo and nonagenarians, and for this analysis, 33 were excluded due to missing cardiac history (*n* = 3), missing risk factors (*n* = 27), or missing BRP mortality-check (*n* = 3) (Fig 1).

### Risk and ACS profile of the elderly ACS patients

The 1036 patients had a median age of 83 yrs (80–100). The age groups 80–84, 85–89, and 90+ consisted of 632, 304, and 100 patients with median ages of 82, 86, and 91 years. A significant difference was observed in the percentage of men across the age groups (Tab. 1): 57% in the 80–84 group, 54% in the 85–89 group, and 42% in the 90+ group. No significant differences were found in cardiac medical history, diabetes mellitus, or smoking. Nonagenarians are characterized by a predominance of women (58%), higher prevalence of hypertension (71%) and lower prevalence of hypercholesterolemia (33%).

**Table 1:** Patient Characteristics

	* 80–84 (N* *=* *632)*	* 85–89 (N* *=* *304)*	* 90+ (N* *=* *100)*	*P‑value*
Median age (min-max)	82 (80–84)	86 (85–89)	91 (90–100)	< 0.001
Male (%)	362 (57.3)	165 (54.3)	42 (42.0)	0.016
*Medical history*				
– Previous MI (%)	151 (23.9)	73 (24.0)	31 (31.0)	0.296
– Previous PCI (%)	219 (34.7)	93 (30.6)	32 (32.0)	0.450
– Previous CABG (%)	69 (10.9)	20 (6.6)	7 (7.0)	0.072
*Risk factors*				
– Smoking (%)	59 (9.3)	28 (9.2)	5 (5.0)	0.356
– DM (%)	163 (25.8)	59 (19.4)	28 (28.0)	0.065
– Hypertension (%)	417 (66.0)	175 (57.6)	71 (71.0)	0.013
– Hypercholesterolemia (%)	267 (42.2)	105 (34.5)	33 (33.0)	0.033
– STEMI (%)	120 (19.0)	72 (23.7)	23 (23.0)	0.213
– NSTEMI (%)	327 (51.7)	175 (57.6)	58 (58.0)	0.174
– Unstable AP (%)	185 (29.3)	57 (18.8)	19 (19.0)	< 0.001
– Multivessel disease (%)	252 (53.7)	107 (57.5)	25 (69.4)	< 0.001
*Laboratory*				
– Hb at adm	8.20 (7.60–8.80)	7.90 (7.10–8.58)	7.80 (6.90–8.40)	< 0.001
– Lowest Hb during adm	8.10 (7.40–8.80)	7.80 (7.00–8.50)	7.80 (6.70–8.30)	< 0.001
– CRP at adm.	2.40 (0.90–8.20)	3.20 (1.00–11.00)	4.00 (0.80–12.25)	0.170
– highest Troponin during adm	1105.0 (5–18,380)	2277.0 (133–18,380)	2445.5 (133–11,333)	0.086
– highest eGFR during adm	64.0 (52.0–79.0)	60.0 (46.0–73.5)	53.0 (40.0–62.3)	< 0.001
CAG (%)	469 (74.2)	186 (61.2)	36 (36.0)	< 0.001
PCI (%)	356 (56.3)	149 (49.0)	30 (30.0)	< 0.001
PCI + CABG (%)	365 (57.8)	149 (49.0)	30 (30.0)	< 0.001
CABG (%)	9 (1.4)	0 (0.0)	0 (0.0)	0.01**

No differences were seen between the prevalence of STEMI and NSTEMI. However, unstable angina pectoris (UAP) was more prevalent in the 80–84 group at 29%, compared to both 19% in the 85–89 and 90+ groups.

### Biochemistry of elderly ACS patients

Biochemical values of the most important determinants of frailty and outcomes are as follows. Octogenarians had the highest median e‑GFR during admission of 64.0 (52.0–79.0) ml/min in the 80–84 group to 60.0 (46.0–73.5) ml/min in the 85–89 group. Nonagenarians had a reduced e‑GFR of 53.0 (40.0–62.3) ml/min (*p* = < 0.001). The median admission hemoglobin (Hb) levels in octogenarian groups were 8.20 (7.60–8.80) and 7.90 (7.10–8.58) mmol/l, while 7.80 (6.90–8.40) mmol/l in nonagenarians (*p* = < 0.001). The median CRP and peak troponin‑I values did not differ between the 3 patient groups.

#### Invasive procedures in elderly

The incidence of STEMI (19% vs 24% vs 23%) and NSTEMI (52% vs 58% vs 58%) was comparable between the age groups. A declining trend for invasive strategy was seen at higher age; CAG was performed in 74% of the 80–84 group, 61% of the 85–89 group, and 36% of the 90+ group. One vessel disease was found to be comparable between the octo and nonagenarians. The rate of PCI also dropped with advancing age: 356 (56%) in the 80–84 group, 149 (49%) in the 85–89 group, and 30 (30%) in the 90+ group (*p* = < 0.001), due to extensive high-risk 3 vessel coronary artery disease and risk of in-cathlab mortality. An invasive urgent CABG strategy was hardly performed in the 80+ cohort (1.4%), and only in those under 85 yrs. Primary PCI was performed in 85% of STEMI cases.

#### Complications in elderly

In-hospital complications did not differ between the groups (Tab. 2). BARC type 3a bleeding occurred in 12 patients (1.9%) in the 80–84 group and 7 patients (2.3%) in the 85–89 group, no type 3a bleedings in the 90+ group were observed, resulting in no significant difference. In-hospital cerebrovascular events were rare and occurred in 1 (0.2%) patient in the 80–84 group and 2 (0.7%) patients in the 85–89 group. In-hospital mortality rates were 2.4% (80–84), 4.6% (85–89) and 3.0% (90+).

**Table 2:** Outcomes and hazard ratios for mortality at 90 days, initial invasive versus optimal medical therapy

	80–84 (*N* = 632)	85–89 (*N* = 304)	90+ (*N* = 100)	*P*–value
Initial ICU admission (%)	10 (1.6)	3 (1.00)	1 (1.00)	0.707**
*Complications*				
– Secondary ICU admission (%)	4 (0.6%)	0 (0.00)	0 (0.00)	0.137**
– CVA (%)	1 (0.2%)	2 (0.66)	0 (0.00)	0.360**
– BARC type 3a (%)	12 (1.9)	7 (2.30)	0 (0.00)	0.297
Re-admission with ACS within 30 days (%)	13 (2.1%)	12 (3.9%)	4 (4.0%)	0.194
In hospital mortality	15 (2.4%)	14 (4.6%)	3 (3.0%)	0.201**
All-cause death at 90 days (%)	39 (6.2%)	42 (13.8%)	20 (20.0%)	< 0.001
	*HR (95% CI interval) until 90 days*			
80–84	0.443 (0.189–1.036) 0.459 (0.197–1.068) 0.159 (0.030–0.849)			0.060
85–89				0.071
90+				0.031

* = independent-samples Kruskal-Wallis test, ** = Likelihood-ratio

Readmission rates for recurrent ACS within 30 days were low: 2.1% in the 80–84 group, 3.9% in the 85–89 group and 4% in the nonagenarian group. No significant differences between the OMT and the invasive group were observed. In the NSTEMI group a significantly lower re-admission rate for ACS at 30 days was seen in the invasive group (1.4%) versus the OMT group (4.3%).

**Fig. 2:**
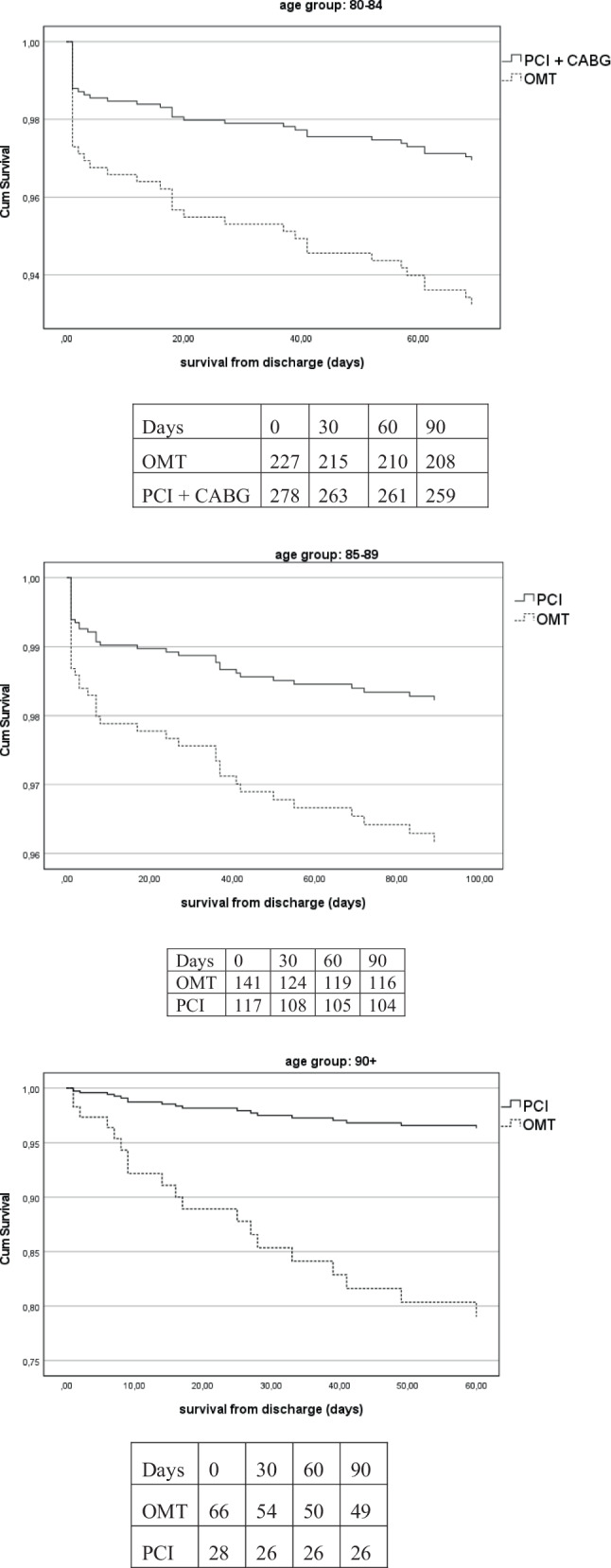
Corrected survival analyses per age group

At 30 days, mortality rate differs: 4% in the 80–84 group and 10% in the 85–89 group (*p* = < 0.001). It was 15% in nonagenarians (*p* < 0.001) (Fig. 2).

In univariate analysis, highest CRP levels during admission are associated with worse mortality outcome HR = 1.012 (1.007–1.017) (*p* = < 0.001) in 80–84 yrs. The highest CRP and lowest Hb levels during admission were associated with a better mortality outcome HR: 0.770 (0.598–0.994) (*p* = 0.044) in 85–90 yrs. A higher eGFR during admission is associated with better mortality outcomes HR: 0.951 (0.919–0.985). By multivariable correction, this is ruled out.

At 3 months, a trend towards a better outcome in all-cause mortality was seen in the invasive group in the age groups: 80–84: HR 0.44 (0.19–1.04) (*p* = 0.06), 85–89: HR 0.46 (0.20–1.07) (*p* = 0.07) and significant better in 90+: HR 0.16 (0.03–0.85) (*p* = 0.03).

## Discussion

In this all-comer consecutive elderly ACS patient’s cohort, 17% were octo and nonagenarian patients with a median age of 83 (80–100)years. Nonagenarians differ in risk profile from octogenarian and are characterized by a predominance of women (58%), higher prevalence of hypertension (71%) and lower prevalence of hypercholesterolemia (33%). At 30 days, mortality rates differ within the 2 octogenarian groups: 4% in the 80–84 group and 10% in the 85–89 group (*p* = < 0.001) and rising to 15% in the nonagenarians (*p* < 0.001). Our findings indicate that favorable outcomes may be most prominent in patients with NSTEMI, although beneficial effects may also be observed in those with STEMI. Procedure-related complications in octo and nonagenarians in whom therapy selection was based on frailty are rare.

### All-comer patients

The current paper includes all-comer octo and nonagenarians who presented with an acute coronary syndrome. These consecutive patients represent current elderly cardiology practice and are significantly older than reported earlier trials [4–12].

Although the 80–84 and 85–90 years cohorts did not differ much with respect to risk factors or cardiovascular history, outcome parameters were significantly worse in the 85–90 years cohort; 30-day mortality (4% vs 10%; *p* < 0.001). Further studies are mandatory to confirm our findings and establish tailored criteria for clinical decision-making.

### EHR data registration

The use of electronic health records are current standard in clinical practice. These EHRs are largely structured data-entry driven and may therefore be suitable to use for research and clinical quality projects and audits. Patient records represent the real-world clinical decision-making process as well as guideline adherence. Our current study shows the additive value of accurate EPD registration by the cardiovascular doctor and nursing team, where teams can prioritize which parameters (e.g. risk-scores, frailty scores, medical history) are structurally entered in the patients’ records and can be moderated in research of quality analyses. Data extraction of the structurally entered data has been implemented in all electronic health record systems and may be represented in dashboard formats. Subsequent systematic review of the data enriches the knowledge of the bedside team and may improve the process of clinical decision-making by reviewing the results at a group level. Next level, structured data entry in an EHR will enable the introduction of EHR-based Randomized Controlled Trials.

#### Frailty in ACS

Frailty is a key factor in determining the prognosis of elderly patients, particularly in critical conditions like (N)STEMI. It is a clinical syndrome characterized by increased vulnerability and reduced physiological reserve and strongly associated with poorer outcomes, including higher complication and mortality rates, longer hospital stays, and more functional decline. This association persists regardless of age or comorbidity [13].

The age-associated activation of inflammatory responses and decline of androgen hormone upset the balance between catabolic and anabolic stimuli, leading to the decline in muscle mass and composition known as sarcopenia [13]. Biologic pathways may lead to the clinical syndrome of frailty with slow walking speed, weakness, weight loss, inactivity and exhaustion. The pathobiology of frailty shares commonalities in biomarkers like hs-CRP, interleukin‑6, hemoglobin, glomerulo-filtration rates, and troponins.

Frailty assessment provides crucial prognostic information that can influence treatment decisions. There are various tools to assess frailty, each differing in time, domains, and methods used. Of these tools, the Clinical Frailty Scale (CFS) seems appropriate for the acute setting due to its simplicity and ability to quickly assess frailty [14, 15]. Emerging methodologies, such as biomarkers and advanced imaging techniques, offer promising avenues for more objective and quantifiable measures of frailty [16].

However, clinicians often rely on subjective clinical judgement and simpler tools during the acute phase. These tools allow clinicians to quickly assess frailty and predict outcomes, helping to guide treatment decisions and improve patient care. The choice of tool should depend on the patient’s condition, the clinical environment, and the available time for assessment.

Our study indicates that rapid clinical judgment, such as the “eyeballing” approach, may be feasible in the acute setting of (N)STEMI when considering invasive strategies for octogenarians and nonagenarians. This method, coupled with skilled operators, may then result in favorable outcomes. Ultimately, it is the combination of this clinical assessment and expert intervention that determines the success of treatments for these elderly patients.

#### Complications

A selected invasive strategy did lead to a few complications (BARC type 3a bleeding, CVA, or secondary ICU admission). Previous studies on PCI in the elderly show comparable results [4, 5, 17–19]. Considering PCI in selected elderly can be a sound clinical decision, taking into consideration the patient’s preference, life expectancy, comorbidities, ACS type and troponin levels. Length of stay was comparable in all groups. Readmission at 30 days was infrequent and significantly lower after an invasive strategy for NSTEMI.

PCI of consecutive octo and nonagenarians with NSTEMI benefits also in the 90+ age group. This corresponds with another real-world study including 296 patients over 80 [18]. The SENIOR-RITA trial with 1518 selected patients from 48 sites did not find a significant benefit of an invasive strategy in NSTEMI [19]. All three studies found a low procedural risk while employing this strategy. Comparably, the Netherlands Heart Registry reports a 11% and 3% 30-day mortality in their 2021–2023 cohort of 9147 STEMI and NSTEMI all-comer patients over 80 years of age, respectively [20].

## Limitations

Because of the observational study design, selection bias and confounding cannot be ruled out. Therefore, the effect of the PCI treatment should be interpreted with care, given the wide confidence intervals in some analyses. Furthermore, all information is taken as reported by the treating physicians, like the risk factors and ACS diagnoses. Frailty scores are not available in the current EHR and do not have a one-size-fits-all solution to predict outcome in the acute setting of (N) STEMI. We therefore explored the potential of biomarkers such as e‑GFR, Hb, and CRP to assist frailty assessment in acute cardiac situations. Further work is mandated to assess whether available biomarkers may be seen as a surrogate value for frailty.

## Conclusion

In this consecutive ACS cohort of 6168 patients, a total of 1069 (17%) were octo and nonagenarians. Nonagenarians differ in patient characteristics compared to patients over 80 and 85. At 30 days, the mortality rate differs within the 2 octogenarian groups: 4% in the 80–84 group and 10% in the 85–89 group (*p* = < 0.001) and rising to 15% in the nonagenarians (*p* < 0.001). This all-comer single-center study shows that physicians may be able to appropriately select ACS octo and nonagenarians for an invasive strategy.
